# Comparing the effects of Osteoporosis Prevention Exercise Protocol (OPEP) versus walking in the prevention of osteoporosis in younger females

**DOI:** 10.12669/pjms.312.5990

**Published:** 2015

**Authors:** Rabail Rani Soomro, Syed Imran Ahmed, Muhammad Khan, Syed Shahzad Ali

**Affiliations:** 1 Rabail Rani Soomro, MSPT, Institute of Physical Medicine & Rehabilitation, Dow University of Health Sciences, Karachi, Pakistan; 2 Dr. Syed Imran Ahmed, FCPS, Institute of Physical Medicine & Rehabilitation, Dow University of Health Sciences, Karachi, Pakistan; 3 Muhammad Khan, MSc.PT, Institute of Physical Medicine & Rehabilitation, Dow University of Health Sciences, Karachi, Pakistan; 4 Syed Shahzad Ali, MSPT, Institute of Physical Medicine & Rehabilitation, Dow University of Health Sciences, Karachi, Pakistan

**Keywords:** Osteoporosis, Prevention, Exercise, Bone mineral density, Younger females, Dual-Energy X-ray Absorptiometry

## Abstract

**Objective:**

The aim of this work was to evaluate the effectiveness of Osteoporosis Prevention Exercise Protocol (OPEP) in younger females.

**Methods:**

One hundred young female volunteers aged 20-30 were selected from IPM&R Dow University of Health Sciences. This was a comparative study in which 64 females participants were randomly assigned into two groups (32 in OPEP exercise group and 32 in walking group). The exercise session had three components 1) stretching 2) strengthening 3) high impact weight bearing exercises. Both interventional programs consisted of 3 sessions per week for twelve weeks under the supervision of physiotherapist. Pre and post intervention bone mass density (BMD) was measured on the lumbar spine (L1–L4), hip, femur, and distal forearm by using Dual-Energy X-ray Absorptiometry (DEXA) scan.

**Results:**

After twelve weeks of intervention BMD was found to be statistically insignificant at hip, femur, lumbar spine and wrist (p > 0.05) comparing the post results in the OPEP and exercise group. Moreover BMD at hip, femur, lumbar spine and wrist was unaltered in both groups comparing the results of pre and post intervention. Though significant changes were observed in BMI in the OPEP exercise group (p value =0.010) mean ± standard deviation pre and post found to be 20.2578 ± 3.11123 and 21.0942 ± 3.64203 but no variations in anthropometrics in walking group were found.

**Conclusion:**

The present study highlights the burden of osteopenia in younger females. The Osteoporosis Prevention Exercise Protocol formulated by author was not useful to bring any significant changes in BMD moreover it had no significant effects in comparison to walking group. However additional studies are needed to evaluate the efficacy of Osteoporosis Prevention Exercise Protocol on bone quality with long term effects.

## INTRODUCTION

In Pakistan, the elderly population is speedily increasing with the rise in population growth. Among estimated population of 162 million (2008 statistics) in Pakistan, aged population over 65 years is 5.36 million.^1^ In the country, estimated female population is 70 million among which about 8.4 million are beyond 50 years of age. Because of this massive rise in elderly population, there comes an urgent need for prevention strategies of age related disorders. Osteoporosis is therefore one of the most prevalent age related disorder and a significant Public Health Problem in Pakistan. It has been assumed that since 1990 in Asia there has been remarkable increase in such fractures, and in near future half of the world’s fractures will occur due to osteoporosis in Asia.^2^ Osteopenia or low bone density is getting more common among younger females at the age of 20 to 30 years.

In a local study conducted in Kkyber Pakhtoonkhwa Province (KPK) of Pakistan, 47.7% females were having osteopenia and 24.7% were having osteoporosis.^3^ Another study conducted in Karachi supports these findings where osteopenia was found in 64% of women <30 years and in 55% of women <45 years.^4^ Hence, to overcome this burden in local population it is significant to set up cost effective preventive strategies at younger age, leading to delay in onset and prevention of the disease. Calcium and vitamins D enriched diet; smoking cessation, lesser consumption of alcohol and caffeine are some fundamental preventive strategies that delay the consequence of osteoporosis. Walking 3 to 5 miles a week may possibly improve bone density.^5^ In addition, exercises are emphasized to have maximum effects on bone mineral density when done at younger age because peak bone mass is known to be achieved around the age of 30 years.^4^,^6^ Weight bearing exercises, in which muscles and bones work against gravity bearing the weight of a person, prevents bone loss associated with aging.^6^ In healthy adults, moderate to high impact exercises are highly recommended to preserve the bone density such as jogging, jumping, stair climbing, step aerobics, weight lifting. While low impact exercises have shown minimal effects on BMD such as walking and swimming.^7^

To our knowledge no such exercise program is designed to provide site specific interventions against osteoporosis combined with muscle strengthening and stretching to enhance BMD at young age. Therefore this study was aimed to encourage the applicability of Osteoporosis Prevention Exercise Protocol (OPEP) which is a combined program of high impact weight bearing exercises, strengthening exercises and flexibility exercises altogether. Meanwhile this study is also supposed to evaluate whether this exercise program can be a valuable adjunct to enhance BMD in younger females.

## METHODS

This study was conducted at Institute of Physical Medicine and Rehabilitation Dow University of Health Sciences Karachi between August 2011 and January 2012 after the approval of Institutional Review Board (IRB). Questionnaire/screening Performa was filled by 130 females at IPM&R, asking information regarding their age, height, weight, ethnicity, physical activity and medical influences, occupational history, menstrual status, status of breast-feeding, parity use of hormones, dietary factors including intake of calcium and vitamin D, amount of sun exposure, vitamin or mineral intakes and consumption of adequate amount of milk/yogurt/cheese daily and status of coffee and tea intake daily. Informed consent was taken by each participant in the study after recruiting 100 females from IPM&R. They were explained to thoroughly read the consent form and give consent that they are participating by their own will. The inclusion criteria were diagnosed osteopenia, normal menstrual history, non-smoker and no known medication such as steroids, hormonal therapy and vitamin D intake. The exclusion criteria were use of contraceptive pills/injections, pregnancy and breast feeding, cardiovascular, respiratory, musculoskeletal or other diseases that may limit exercises and obese females, adequate consumption of milk and tea more than 4 cups a day. Pre intervention height, weight and Body Mass Index (BMI) were measured. Bone Mineral Density (BMD, g/cm^2^), of these 100 participants was performed on the lumbar spine (L1-L4), left hip, left neck of femur and left distal radius with dual-energy X-ray absorptiometry (DEXA).

The scanning and analyses was done by the same operator. Overall 70 participants were diagnosed with condition osteopenia among which 6 participants refused to participate in further research. The remaining 64 osteopenic participants were equally and randomly assigned into two groups by simple randomization method. Group A=32 was experimental group and performed Osteoporosis Prevention Exercise Protocol (OPEP) consisting of stretching, strengthening and high impact weight bearing exercises and Group B=32 performed walking as control. Both sessions were done under supervision of Physiotherapist for 30 minutes. Intervention was carried out 3 times a week for 3 months duration. Post intervention height, weight, BMI and BMD was measured for comparison with pre intervention data.

### Statistical analysis

SPSS version 16 was used for data analysis. Statistical results are expressed as mean ± standard deviation (S.D) for quantitative data. Paired sample t test was first used to compare the overall difference in pre and post treatment effects on height, weight, BMI and BMD of all the study participants. Additionally, this test was also used to compare group A and group B for their pre and post treatment effects. Independent sample t-test was used to determine the mean difference and level of significance in post treatment effects on weight, height, BMI and BMD of both group A and group B. In all tests p value <0.05 was considered statistically significant

## RESULTS

Among 100 young female volunteers, 70 females were diagnosed with osteopenia. Out of 70 females 64 females were selected for further study. The sample size was 64 out of which 8 girls dropped out from the study. The reasons for dropping out were studying for exams and being sick. Fifty-six girls on the whole completed this three months randomized controlled trial out of which twenty-nine were in exercise group and twenty seven were in walking control group. There were no significant differences at baseline in anthropometric measures and BMD measures among both groups however considerable variation in BMD of spine was observed at baseline among both groups. The results showed that there were no statistically significant differences at baseline and post intervention results on BMD of hip, spine, femur and wrist among exercise and walking group (p>0.05), however mean body weight was found to be significantly increased after intervention from baseline p=0.034 comparing overall results among both groups.

Moreover comparing the effects of exercise and walking among both groups there were no meaningful differences in measurements of BMD before and after interventions. Though significant changes were observed in height, weight and BMI in the exercise group after intervention from baseline but no changes in anthropometrics in walking group were found.

Comparing the post intervention effects among exercise and walking group in Table-I it has been shown that anthropometrics, BMD hip, lumbar spine, femur and wrist among both groups remains to be insignificant.

**Table-I:** Difference in mean and standard deviation pre and post among both groups.

N=56	Baseline	After 3 Months	p-value
Height (m)	1.5444 (0.05801)	1.5916 (0.34127)	0.283
Weight (kg)	48.0484(8.38119)	50.0410 (9.99293)	0.034
BMI (kg/m2)	20.1266(3.15919)	20.4260(4.19818)	0.288
BMD (gm. /cm^2^)					
Hip	0.8866 (0.09665)	0.8881 (0.09208)	0.791
Neck of femur	0.8591(0.10014)	0.8053(0.29403)	0.150
Spine (L1-L4)	1.0717(0.11017)	1.0146(0.30623)	1.35
Wrist	0.5631(0.03565)	0.5135 (0.42785)	0.378

BMD=Bone Mineral Density; BMI=Body Mass Index.

## DISCUSSION

In this study 70% of disease expresses osteopenia to be a significant problem in younger female population. Present study findings are in agreement with previous study where BMD was found to be decreased in 64% of women <30 years.^4^ In both of these studies targeted population suffering from osteopenia was 20 to 30 years. Though, in present study frequent age among females was 20 to 23 years and the result showed that this particular age group is more prone to suffer from low bone density or osteopenia. Furthermore, in present study participants in the sample were graduating students and it is a single center study. Some reviews and meta-analysis have suggested that peak bone mass is known to be achieved during childhood and adolescence.^8^ Therefore this study was done to discover the benefits of exercise in younger females. Walking is a form of low impact exercise and OPEP is a combination of different exercises. Present study showed that OPEP exercise protocol is not significant in enhancing BMD in younger females with comparison to walking. After twelve weeks of intervention period, this study revealed no statistically significant increased BMD in OPEP group at any of the weight-bearing site. However significant changes on BMI were seen in the OPEP group while no significant alteration in BMI was seen in control group. This is in contrast to Kerr *et al*.^9^ study which reported that strength training and progressive loading shows 0.9% increase in BMD in the exercise group at the hip site while no difference in forearm, lumbar and whole body between exercise and control group were seen. The differences in the results may be due to exercise protocol applied, duration of the study, age group chosen, and the method and site used to determine bone status. In another study by Prince *et al*.^10^ weight bearing exercise along with calcium supplementation showed significant differences on BMD of hip, spine, femoral neck and radius. This significant alteration in multiple sites BMD could be due to usage of calcium supplementation in the study while in the present study only exercise effects have been observed. Another recent population-based study on 25 years old 1061 women showed that recreational physical activity; jogging and weight bearing exercises improved BMD significantly in younger age women.^11^ In our study and this study population was same but they have used large sample size, which could have impact on the results.

Conversely to present study results, structured exercise programs including high impact weight bearing exercise have shown positive effects on BMD mostly on lumbar spine and femur.^12^-^14^ The samples in these studies were post-menopausal women but in this study sample were young osteopnenic females. Rarely few studies are done to show the effects of exercise among young or pre-menopausal women with osteopenia. Fried Lander *et al*.^15^ has reported that a 2- year high-impact exercises period have produced significant positive differences in BMD between the exercise and stretching groups for femoral trochanteric (2.3%), femoral neck (2.4%), calcaneal (6.4%) measurements and spinal trabecular (2.5%) among younger females at the age of 20-35 years. However, compared to this study our study includes only three months of exercise program, which is relatively short duration. Moreover, a systematic review conducted by Wallace and Cumming^16^ has indicated positive effect on the lumbar spine in both high-impact and non impact exercises, but only high-impact exercise brought a positive effect on the femoral neck.

OPEP is an exercise protocol, which focuses on stretching session of postural muscles and strengthening of abdominals and back extensors to improve the axial loading on skeleton. Meanwhile in impact exercise muscle forces may also play some role rather than just having effects through weight-bearing on bones. Despite the addition of these exercises no differences were seen on the bone mineral density however, no outcome tool was used to measure the strength of the muscles or calculate muscle bulk. Although some studies also focuses on strengthening exercises for improving muscle strength and power so as mechanical load is not directly delivered on the skeleton.^17^ This study was a short duration study and numerous studies have also asserted that exercise of quite short interval did not enhance BMD, but decline in BMD were reported in non-exercise group.^18^ However in current study no decline was declared in the OPEP and walking groups.

Habibzadeh *et al*.^19^ has suggested that 30 minutes of walking exercise at the range of 50%-75% maximum heart rate was not enough to bring significant change in the BMD in thin girls. This study has strong correlation with the current study considering the population of 20 young girls who were diagnosed osteopenic at the age of 20-25 years. In the study low BMI is also shown to be associated with low body weight and hence thin girls are proved to be more prone to risk of osteoporosis. In this ultimate study 30 minutes of walking for three times per week for the duration of twelve weeks was not enough to bring significant improvement in bone density. A prior study has suggested that three month walking program did not bring significant changes in bone mineral content.^20^ However, some long-term studies also support walking to be non-effective in improving BMD.^21^ The apparently conflicting results may be due to characteristics of the volunteers participating (e.g., age, nutrition, and hormonal status) or the selection of the study samples and additionally differences in the type, intensity, frequency, or duration of exercise may also have affected the results. Body weight changes have strong linkage to BMD changes in women.^19^ In this study significant weight and BMI changes in the OPEP group have been observed but no significant correlation of BMD and BMI has been seen in the results. Therefore this can be assumed that the weight changes might be due to increased food intake by the participants within the study duration. In this study no diet control prescription was given to the participants and no diet measurement diary was filled to keep the record of food intake. In contrast to our study, few studies have successfully monitored the weekly record of the dairy food intake taken by the participants.^22^

### Limitations of Study

The outcome measure tool DEXA scan was used, although it is standard tool for measurement of BMD, various other tools could have been used and there was no outcome measure used to see fitness among students. Our study was bounded to single center setting and the demerit we had with the single center setting was we had some specific age groups limited between 20-23 more participants and less participants between age group of 24-30 because all students were fresh and graduate students. The duration of study was short. If it would have been more than six months we had expected some significant results. Male participants could also have been part of the study.

## CONCLUSION

Both interventions are effective and Osteoporosis Prevention Exercise Protocol did not produce significantly better changes in BMD as compared to walking group. The duration of this study was too short and further studies are needed to evaluate the efficacy of Osteoporosis Prevention Exercise Protocol on bone quality with long-term effects.

## References

[1] Raza SA (2011). Endocrinology in Pakistan: Transcending in care of endocrinological disorders Indian. J Endocrinol Metab.

[2] Suzuki T (2001). Risk factors for osteoporosis in Asia. J Bone Miner Meta.

[3] Arif M, Inam M (2006). Frequency of osteopenia & osteoporosis in different parts of North West Frontier Province. J Postgrad Med Ins.

[4] Jaleel R, Nasrullah FD, Khan A (2010). Osteopenia in the younger females. J Surgery Pakistan Int.

[5] Karlsson MK, Nordqvist A, Karlsson C (2008). Physical activity increases bone mass during growth. Food. Nutr. Res.

[6] Warburton DER, Nicol CW, Bredin SSD (2006). Review, Health benefits of physical activity: The evidence. CMAJ.

[7] Forwood MR, Larsen JA (2000). Exercise recommendations for osteoporosis. A position statement of the Australian & New Zealand bone & mineral society. Aust Fam Physician.

[8] Nikander R, Sievanen H, Heinonen A, Daly RM, Uusirasi K, Kannus P (2010). Targeted exercise against osteoporosis: A systematic review and meta-analysis for optimizing bone strength throughout life. BMC Med.

[9] Kerr D, Ackland T, Malsen B, Prince R (2001). Resistance training over two years increases bone mass in calcium-replete postmenopausal women. J Bone Miner Res.

[10] Prince R, Devine A, Dick I, Criddle A, Kerr D, Kent N (1995). The effects of calcium supplementation (milk powder or tablets) & exercises on bone density in postmenopausal women. J Bone Miner Res.

[11] Callreus M, McGuigan F, Ringsberg K, Akesson K (2012). Self-reported recreational exercise combining regularity & impact is necessary to maximize bone mineral density in young adult women. Osteoporos Int.

[12] Bravo G, Gauthier P, Roy PM, Payette H, Gaulin P, Peloquin L (1996). Impact of 12 month exercise on the physical & physiological health of osteopenic women. J Am Geriatr Soc.

[13] Grove KA, Londeree BR (1992). Bone density in postmenopausal women: High impact vs. low impact exercise. Med Sci Sports Exerc.

[14] Chow R, Harrison JE, Notaries C (1997). Effect of two randomized exercise programs on bone mass of health postmenopausal women. BMJ.

[15] Friedlander AL, Genant HK, Sadowsky S, Byl NN, Gluer CC (1995). A two-year program of aerobics and weight training enhances bone mineral density of young women. J Bone Miner Res.

[16] Wallace BA, Cumming RG (2000). Systematic review of randomized trials of the effect of exercise on bone mass in pre and postmenopausal women. Calcif Tissue Int.

[17] Russo CR (2009). The effects of exercise on bone. Basic concepts & implications for the prevention of fractures. Clin Cases Miner Bone Metab.

[18] Kemper HHCG, Baker I, Van Tulder MW, Kostense PPA, Courteix D (2000). Exercise for preventing low bone mass in young males & females (Protocol). Cochrane Database of Systematic Reviews.

[19] Habibzadeh N, Rahmanii-Nia F, Daneshmandi H (2010). The effect of walking exercise on bone mass density in young thin women. World Sport Sci.

[20] Dalen N, Osen JM (1974). Bone mineral content and physical activity. Acta Orthop Scand.

[21] Cavanaugh DJ, Cann CE (1988). Brisk walking did not stop bone loss in postmenopausal women. Bone.

[22] Bloomfield SA (2001). Optimizing bone health: Impact of nutrition, exercise & hormones. J Sports Sci Exchange.

